# Modulating the Aqueous Micellar Reorganization of Sequence-Defined Ionic Peptoid Block Copolymers by Ionizable Monomer Position and Solution pH

**DOI:** 10.1021/acs.macromol.4c02829

**Published:** 2025-02-10

**Authors:** Bailee N. Barrett, Pedram AziziHariri, Vijay T. John, Donghui Zhang

**Affiliations:** † Department of Chemistry and Macromolecular Studies Group, Louisiana State University, Baton Rouge, Louisiana 70803, United States; ‡ Department of Chemical and Biomolecular Engineering, Tulane University, New Orleans, Louisiana 70118, United States

## Abstract

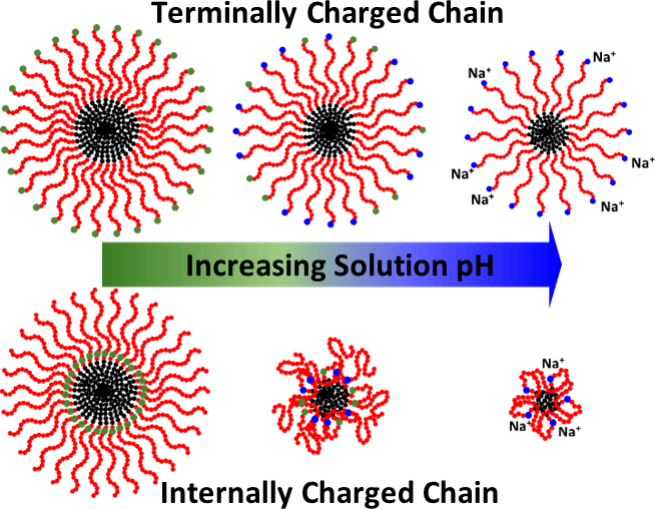

The micellar aggregation of singly charged sequence-defined ionic peptoid block copolymers can be finely tuned by adjusting the position of the ionizable monomer along the chain and varying the solution pH. The pH-induced structural reorganization of these micelles was found to depend on the position of the ionizable monomer along the chain, influencing the balance of the hydrophobic interactions, excluded volume effect, and electrostatic forces (i.e., charge repulsion, solvation of the ionic monomers, counterion association) that govern the micellar structure. As the solution pH increases, positioning the ionizable monomer closer to the junction of the hydrophobic and hydrophilic blocks causes a larger reduction in the micellar size and aggregation number across two distinct regimes. In contrast, placing the ionizable monomer at the terminus of the hydrophilic block results in a smaller reduction in the micellar size and aggregation number over three regimes. This study provides new insights into leveraging the strategic positioning of ionizable monomers to design stimuli-responsive nanoassemblies capable of programmable structural reorganization.

## Introduction

Functional biomaterials composed of self-assembled structures with customizable properties are highly sought after for their potential applications across diverse fields, including drug delivery,^1−4^ biosensing, and bioimaging.^5−7^ Polypeptides play a key role in these efforts by providing a robust framework for designing tunable, synthetic nanoscale structures and showcasing how monomer sequencing influences the structural outcomes and biological functionality. The ionic nature of certain amino acids introduces electrostatic interactions into the system, enhancing the structural stability of these aggregates within their native environments. Additionally, the type of ionic residues (i.e., weak or strong electrolytes) can render the system stimuli-responsive, enabling sensitivity to environmental changes, such as temperature, pH, and ionic strength. A notable example is hemagglutinin, a peptide in the influenza virus that undergoes conformational changes in low pH conditions to facilitate host cell infection.^8,9^ Such natural instances inspire the design of weakly ionic synthetic polymers for pH-responsive materials with adaptable self-assembly in solution, enabling the modulation of electrostatic interactions in response to solution pH variations to control self-assembly behavior.

Amphiphilic polyelectrolytes, particularly ionic block copolymers (BCPs), have been extensively studied for their solution self-assembly properties. These polymers contain both hydrophilic and hydrophobic segments that self-organize into diverse aggregate geometries in aqueous solutions to reduce interfacial tension between the hydrophobic segments and the surrounding solvent. The ionic monomers contribute additional stability to the aggregates through favorable interactions with the solvent.^10^ Significant research efforts have been focused on investigating the pH responsiveness of solution aggregates formed from ionic BCPs synthesized via various controlled polymerization techniques.^11−13^ These aggregates have been found to adapt to changes in electrostatic interactions through global structural reorganization, including changes in aggregation number,^14−16^ core–corona inversion,^17^ or aggregate agglomeration.^18^ Alternatively, in kinetically trapped aggregates, which often feature a large hydrophobic core, the structural response to pH change is primarily limited to size adjustments through the shrinking or stretching of coronal chains, while aggregation numbers remain constant.^19−21^ Irrespective of the self-assembled aggregate’s structure, their constituent materials often exhibit compositional variation due to the statistical nature of the polymerization method used for their preparation. Conventional polymerization techniques produce materials with dispersity in both block and chain lengths, obfuscating the effects of monomer sequencing—particularly the number and position of ionizable monomers—on the resulting self-assembled structures.

Advances in synthesis have enabled access to nonbiological, sequence-defined polymers with discrete chain lengths and customizable chemistry, allowing precise control over monomer sequences and paving the way for new materials with tailored functions.^22−25^ Sequence-defined ionic polymers offer opportunities to explore how the number and positioning of ionizable monomers along the chains influence their solution association and stimuli-induced structural reorganization. Ionic peptide amphiphiles with varied architectures and defined monomer sequences have demonstrated responsiveness to solution pH^26−29^; some of these structural responses include pH-induced disassembly of aggregates,^30^ geometrical shape changes,^31^ and formation of topographical features.^32^ Due to the stereogenic centers and secondary amide groups along the backbone, peptides have a strong tendency to adopt secondary structures (e.g., β-sheet or α-helix) stabilized by extensive hydrogen-bonding interactions. Consequently, understanding the roles of sequence-encoded electrostatic interactions in guiding the aggregation and structural reorganization of ionic peptide assemblies is nontrivial.

Sequence-defined peptoid polymers, or *N*-substituted polyglycines, represent a promising class of peptidomimetic materials for exploring how sequence-encoded electrostatic interactions can be leveraged to modulate solution self-assembly and influence material properties.^25,33−36^ The *N*-substitution eliminates the backbone hydrogen bonding and stereogenic centers, thus allowing for isolation of the sequence-encoded electrostatic effect on the solution aggregation of ionic peptoid polymers. Previous studies have shown that the micellar size and internal organization of these sequence-defined BCPs can be tailored by the molecular design of peptoid chains.^37−40^ A deeper understanding of how to use this design, in conjunction with various stimuli, to achieve predictable and tailorable structures is still needed.

**Figure 1:**
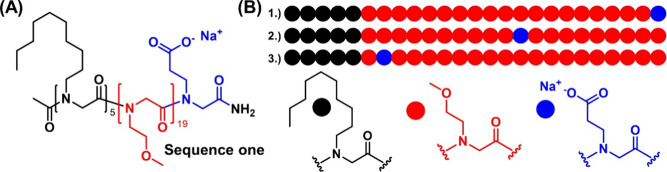
(A) Chemical structure of sequence one of the ionic peptoid BCPs and (B) schematic depiction of the monomer sequences of the ionic peptoid BCPs (1–3).

In this contribution, we investigated how ionizable monomer position along a chain, in conjunction with solution pH, can be used to control the micellar aggregation of amphiphilic, sequence-defined ionic peptoid BCPs by using a combination of scattering and microscopy techniques. In our previous work, we designed and synthesized a series of peptoid diblock copolymers, discovering that repositioning a single ionizable monomer along an otherwise neutral, molecularly equivalent chain led to systematic changes in micellar size and internal structure, such as water and polymer distribution and packing.^37−39^ The relatively short hydrophobic segments and high water content in these peptoid micellar cores suggest the potential of dynamic micelles capable of undergoing global structural reorganization. Herein, we introduce an additional layer of control over micellar aggregation by adjusting the solution pH, leveraging the weak acid functionality of the ionic monomer. Systematic increases in pH led to progressively smaller micellar aggregates for all three micelles (Figure 1), irrespective of ionizable monomer placement. However, the extent of size change and the manner in which increased electrostatic repulsion is accommodated were found to depend directly on the position of the ionizable monomer. This work contributes to the ongoing effort to establish design principles for creating synthetic nanoscale structures with precise and customizable properties.

## Results

We synthesized three sequence-defined ionic peptoid diblock copolymers (Figure 1) using a previously reported solid-phase submonomer method to obtain polymers with discrete chain length (*Đ* = 1.00) and precise monomer sequence.^39^ Each sequence in the library is chemically equivalent, consisting of 25 total monomer units (DP = 25). The hydrophobic block is composed of five neutral *N*-*n*-decyl glycine monomers (D), and the hydrophilic block is composed of 20 total monomers, 19 neutral *N*-2-methoxyethyl-glycine monomers (ME), and one ionic *N*-2-carboxyethyl-glycine monomer (CE). Variations in the sequences arise from the systematic repositioning of the ionizable monomer along the hydrophilic block. The polymer composition has been verified by MALDI-TOF MS spectrometry (Figure S1), and the purity level was found to be >99%, evidenced by HPLC analysis (Figure S2). We have previously shown that these peptoid BCPs can form micelles in aqueous solution at pH ∼ 9 with a critical micellar concentration in the 0.02–0.05 mg/mL range.^39^ The resulting micellar size can be effectively tuned by repositioning the ionic monomer, where progressively smaller aggregates were obtained by moving the ionic monomer closer to the hydrophobic/hydrophilic block junction.^37,39^ In this study, it was further shown that the micellar structure can be tailored by controlling the solution pH, effectively modulating the extent of ionization of the carboxylic acid groups on the CE monomers. The position of the ionizable monomer serves to modulate the relative contribution of electrostatic interactions to the overall free energy that dictates the micellar structure.

The range of solution pH values (2.10 < pH < 10.11) for this study was selected to capture the changes in micellar size as the carboxylic acid groups present in the ionic monomers shift from protonated (COOH) to deprotonated (COO^–^). We previously determined the p*K*_a_ value for each of the aggregated peptoid sequences to be 5.04 ± 0.02, 5.37 ± 0.08, and 5.63 ± 0.03 for sequences one, two, and three, respectively.^39^ Thus, at low solution pH values, we expect high carboxylic acid content and low carboxylate ion content; as the solution pH is increased, we expect the carboxylate ion content to continually increase until all ionic monomers are deprotonated. All peptoid BCP solutions were prepared by direct dissolution of the polymer in preboiled ultrapure water, followed by adjustment of the solution pH by additions of either aqueous NaOH or HCl solutions. The solutions were then heated at 70 °C for 2 h to facilitate micellar equilibration, followed by cooling to room temperature prior to any measurement. Solution pH values are presented as ΔpH values, where ΔpH = pH – p*K*_a_.

### Dynamic Light Scattering Analysis

**Figure 2:**
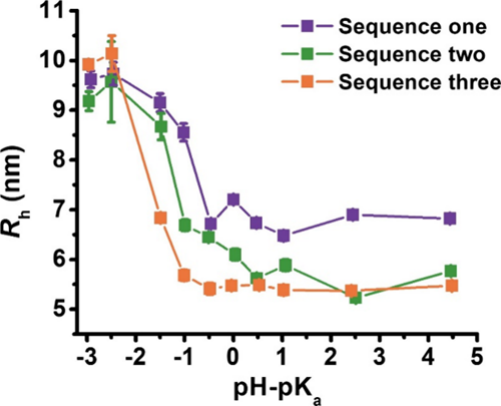
Hydrodynamic radius (*R*_h_) as a function of pH-p*K*_a_ (ΔpH; i.e., increasing ΔpH corresponds to increasing solution pH) for peptoid BCP micelles. Values were obtained from cumulant fitting of DLS correlation data, and error bars were obtained from duplicate measurements.

Dynamic light scattering (DLS) of the micellar solutions shows that the ionizable monomer position, in conjunction with solution pH, can be used to modulate the hydrodynamic size of the micellar aggregates (Figure 2). Irrespective of the ionic monomer position, the largest micelles are observed when the solution pH is low (i.e., the lowest carboxylate ion content). Increasing the solution pH results in an initial decrease in the micellar size until a plateau is reached when ΔpH > 0 (corresponding to >50% ionization of the ionic monomer), at which point the micelles maintain their size despite further increases in solution pH. The position of the ionizable monomer has been found to influence the magnitude of hydrodynamic size changes that the micelles undergo across the studied pH range. As the ionizable monomer is repositioned closer to the hydrophobic/hydrophilic block junction, the micelles undergo a more significant hydrodynamic size change as solution pH is increased. Sequence one micelles, where the ionizable monomer is on the hydrophilic block terminus, exhibits a smaller net decrease in micellar size when compared to those micelles where the ionizable monomer has been repositioned into the coronal interior (sequences two and three). At high solution pH values, where the extent of ionization is greatest, the micellar size shows a clear dependence on ionizable monomer position; as the ionic monomer is repositioned closer to the hydrophobic/hydrophilic block junction, the hydrodynamic size of the micelles becomes progressively smaller.

### Small-Angle Neutron Scattering Analysis

**Figure 3:**
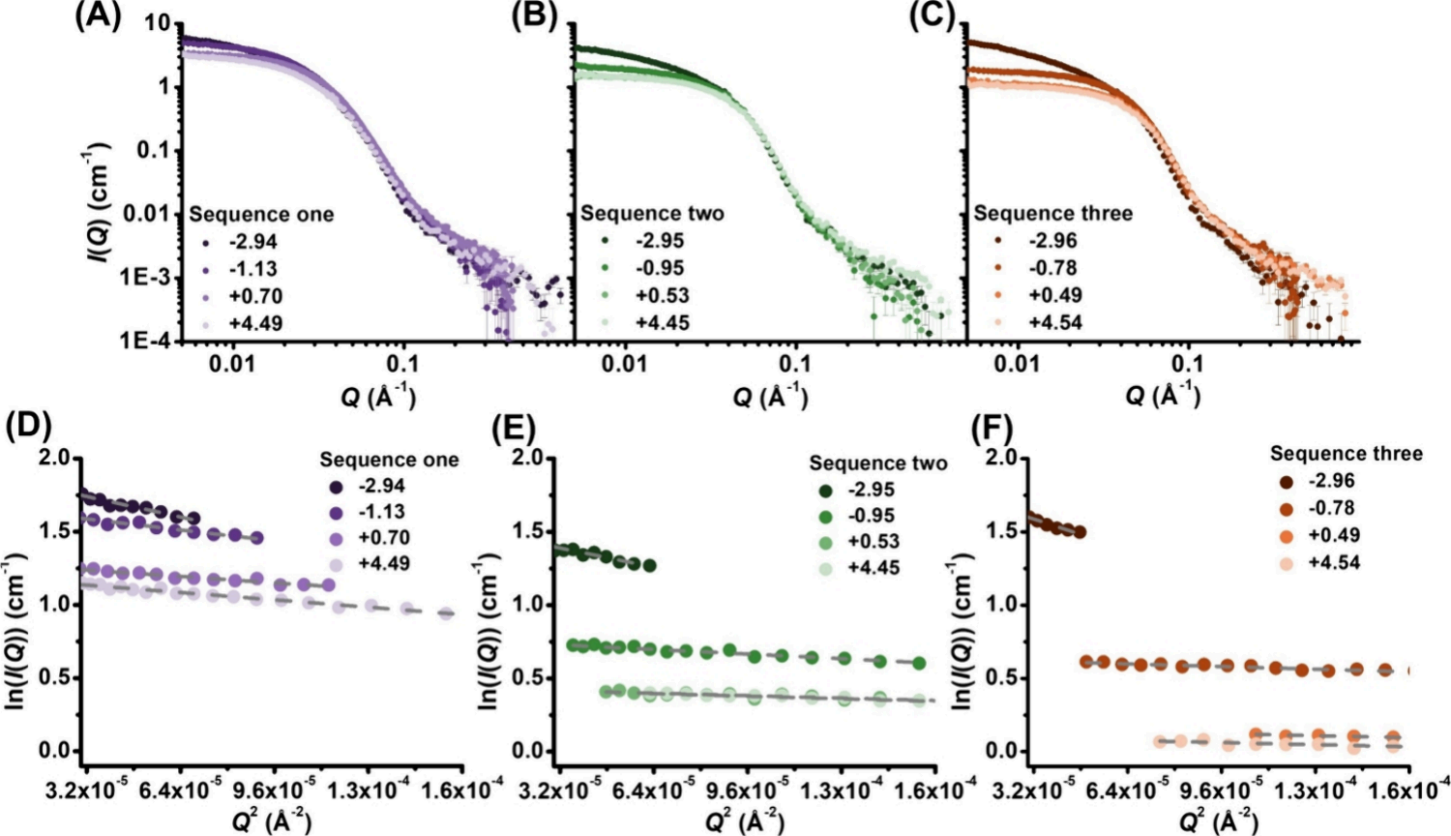
(A–C) Absolute scattering intensity profiles of peptoid BCP micelles (sequence one (A), sequence two (B), sequence three (C), Figure 1) at four of the solution ΔpH values; the entire set of 10 is provided in the Supporting Information (Figures S3–S5). (D–F) Representative region of the Guinier plot for each sequence (sequence one (D), sequence two (E), sequence three (F), Figure 1) at four measured solution ΔpH values; the gray dashed line represents the linear fit of the data. All 10 Guinier plots are shown in Figures S3–S5. Decreasing the color intensity represents a higher solution pH (i.e., higher values of ΔpH).

**Figure 4:**
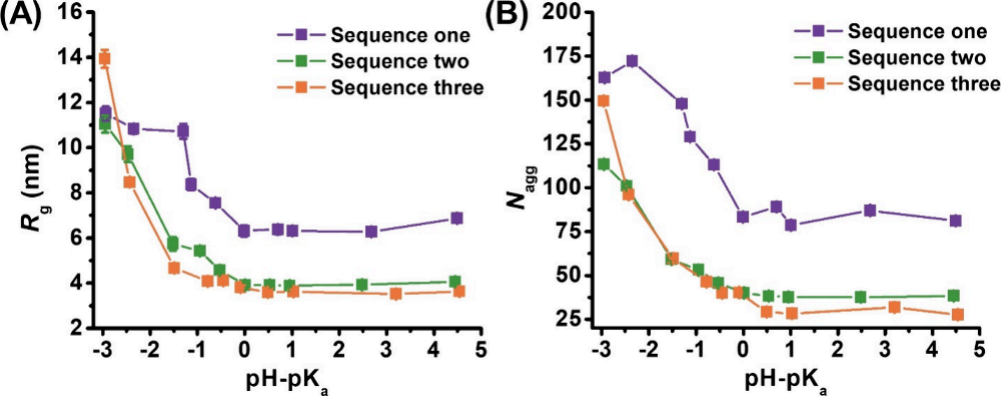
(A) Radius of gyration (*R*_g_) and (B) aggregation number (*N*_agg_) as a function of pH-p*K*_a_ (ΔpH; i.e., increasing ΔpH corresponds to increasing solution pH) for peptoid BCP micelles. Values of *R*_g_ and *N*_agg_ were obtained from Guinier analysis of the respective SANS profiles, and error bars are from data fitting by Guinier analysis.

To further characterize the structural changes of the micelles as a function of solution pH, small-angle neutron scattering (SANS) experiments were conducted on a series of peptoid BCP micellar solutions, and the resulting scattering profiles (*I*(*Q*)) are shown in Figure 3. Qualitatively, as the solution pH is increased, there is a progressive decrease in the scattering intensity at low *Q* values (0.005 < *Q* < 0.02 Å^–1^), indicating a notable decrease in the micellar aggregation number (*N*_agg_) as the extent of ionization is increased. The low *Q* scattering intensity is nearly invariant with *Q* or exhibited a weak power law dependence on *Q* (i.e., *I*(*Q*) ∼ *Q*^α^) with an exponent (α) in the −0.05 to −0.519 range (0.005 < Q < 0.01 Å^–1^). Thus, Guinier approximation of the SANS data (using criteria *R*_g_ × *Q* < 1.0) was applied to obtain quantitative information regarding the micellar aggregation number (*N*_agg_) and radius of gyration (*R*_g_). The results are shown in Figure 4, and the details of the analysis are provided in the Supporting Information.

The dependence of *N*_agg_ and *R*_g_ on the solution pH was found to parallel each other and are consistent with those observed for the micellar hydrodynamic radius (*R*_h_) determined by DLS analysis. Decreasing micellar sizes (both in *N*_agg_ and *R*_g_) were observed with increasing solution pH until ΔpH > 0, where the micellar dimensions become resistant to changes with further solution pH increases. The trends show a clear correlation to ionizable monomer position, as progressively smaller aggregates form as the ionizable monomer is repositioned further into the micellar interior. Also of note is the apparent plateau seen at low solution pH values (ΔpH < −1.25) for sequence one micelles, and the absence of this plateau in the pH range studied for sequences two and three micelles. When the ionizable monomer is positioned at the terminus of the hydrophilic block, the micellar dimensions exhibited minimal change with increasing solution pH (at ΔpH < −1.25). However, repositioning the ionizable monomer into the hydrophilic block interior (sequences two and three) results in a steady decrease in the micellar dimensions (*R*_g_ and *N*_agg_). These results are indicative of dynamic micelles that undergo structural reorganization by changing the aggregation number to accommodate the increasing electrostatic interactions in the micelles with increasing solution pH.

### Zeta Potential Measurements

**Figure 5:**
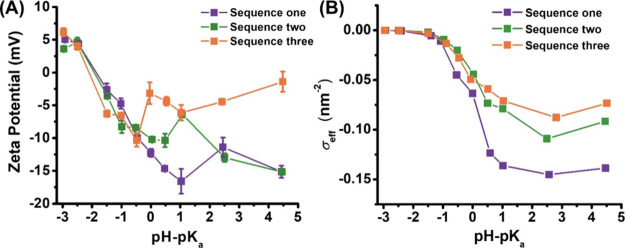
(A) Zeta potential and (B) effective surface charge density as a function of pH-p*K*_a_ (ΔpH; i.e., increasing ΔpH corresponds to increasing solution pH) for peptoid BCP micelles.

The zeta potentials of the micellar solutions as a function of the solution pH were determined by electrophoretic light scattering methods. As shown in Figure 5A, when ΔpH < 0, the zeta potential of all micelle types becomes more negative with increasing solution pH, consistent with increasing extent of ionization and decreasing micellar size. At the lowest ΔpH end (−2 ∼ −3), the zeta potential of all micelle types, regardless of sequence, is approximately +5 mV. At this low ΔpH end, all three sequences are expected to behave similarly as nearly all ionizable CE monomers exist as the protonated carboxylic acid form (COOH) with minimal extent of ionization, resulting in approximately neutral micellar aggregates. As ΔpH was increased, the zeta potential was found to decrease steadily (i.e., become more negative) and plateau at negative values when solution ΔpH > 0; sequence one micelles exhibited the most negative zeta potential and sequence three micelles the least. If these micelles are considered as soft colloids, the zeta potential is indicative of the effective surface charge density of the micelles. Using the micellar hydrodynamic radius (*R*_h_), micellar aggregation number (*N*_agg_), and the extent of ionization (α) at a given pH, we can estimate the effective surface charge density (σ_eff_) of the micelles using the equation: σ_eff_ = (α*N*_agg_)/(4π*R*_h_^2^) by assuming that the micelles are spherical colloids. As shown in Figure 5B, the effective surface charge density exhibited a similar sigmoidal dependence on the ΔpH for all three micellar types. The σ_eff_ value started out low at low ΔpH ends, increased significantly within ca. −1 to 1 ΔpH range, and plateaued at the high ΔpH range. Note that the plateaued σ_eff_ value is the highest for the sequence one micelles, intermediate for the sequence two micelles, and lowest for the sequence three micelles. A clear discrepancy exists between the dependence of the zeta potential on the ΔpH and that of the calculated effective surface charge assuming a spherical colloid model for the micelles. Several factors may have contributed to the observed discrepancy. For instance, when ionic monomers are buried into the micellar interior, as in sequences two and three, they contribute less to the effective surface charge of the micelles.^41^ Counterion association with the micelles can also alter the effective charge density of the micelles. Thus, we attribute the observed dependence of zeta potential on ΔpH > 0 to a combination of factors, including micellar chain packing and conformation, extent of ionization, and counterion association, all of which are modulated by the position of a single ionizable monomer. In addition, the plots of normalized effective surface charge density versus pH-p*K* for these three peptoid micelles were found to be nearly identical (Figure S7). This highlights a self-regulating characteristic of these peptoid micelles attributed to the same ionizable monomers, despite differences in their placement within the micelles.

### Cryogenic Transmission Electron Microscopy Analysis

**Figure 6:**
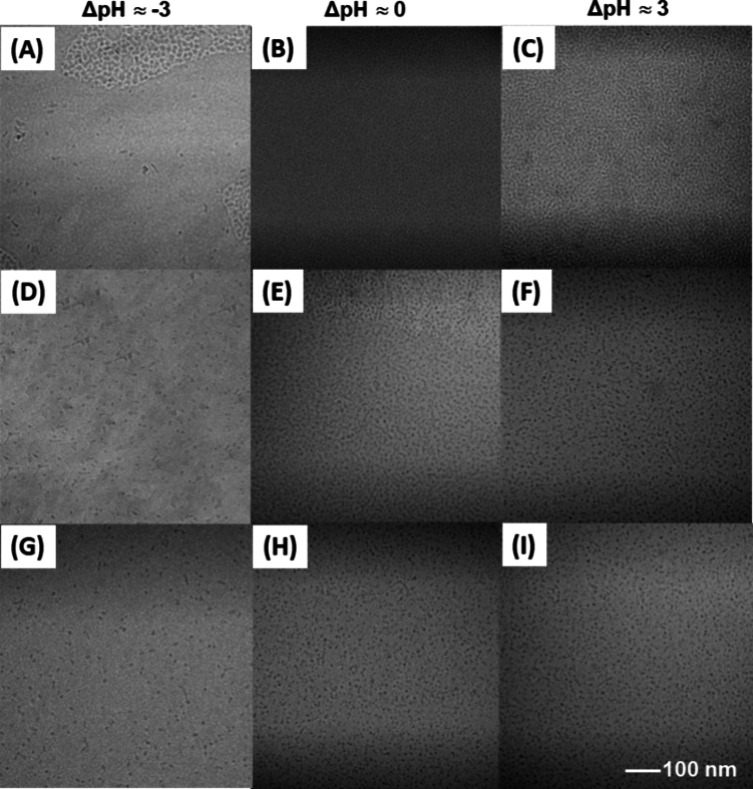
Representative cryoTEM images of sequence-defined ionic peptoid BCP micelles (sequence one (A–C), sequence two (D–F), sequence three (G–I), Figure 1) at three different solution ΔpH values (i.e., ΔpH ≈ −3, 0, and 3). The corresponding histogram analyses of the micellar radius distribution are shown in Figure S8.

To further verify the micellar morphology and dimension, we conducted cryogenic transmission electron microscopy (cryoTEM) analysis of the micellar solutions of sequence one, two, and three peptoids at three selected pH ranges (low: ΔpH ∼ −3, intermediate: ΔpH ∼ 0, and high: ΔpH ∼ 3). CryoTEM analysis revealed spherical micelles with comparable mean micellar radius (*R*_m_) between 3.7 ± 0.3 and 4.1 ± 0.4 nm for all peptoid sequences at the intermediate-to-high pH range (Figures 6 and S8 and Table S2). At a low pH range, slightly ellipsoidal micelles with mean aspect ratios of 1.2 ± 0.2–1.3 ± 0.2 and mean micellar radii (*R*_m_) of 4.8 ± 0.6–6.3 ± 0.8 nm were observed. The micellar radius (*R*_m_) determined by cryoTEM analysis is generally smaller than the hydrodynamic radius (*R*_h_) and radius of gyration (*R*_g_) obtained by DLS or SANS analysis (Figure S8 and Table S2), which is likely due to solvent penetration into the micellar corona, resulting in reduced electron contrast between the micelle and solvent background in the cryoTEM images.

## Discussion

Ionic micelles can accommodate increasing electrostatic interactions by various mechanisms, depending on the nature of the aggregate formed. Kinetically trapped, or “frozen”, micelles have been shown to mitigate the effects of electrostatic interactions through chain stretching within the coronal domains.^19−21^ In contrast, the translational freedom of dynamic micelles allows for alleviation through global structural reorganization.^14,16,42−44^ In both cases, associations between small molecule counterions and oppositely charged micelles can effectively screen the electrostatic repulsion of the ionic monomers within the micelles. As all three micellar types in this study exhibit notable changes in aggregation number with increasing solution pH, it is evident that these micelles are dynamic and capable of global structural reorganization, involving changes of chain packing and conformation inside the micelles. We attribute the changes in micellar size to the interplay of the hydrophobic effect, excluded volume interactions, and electrostatic forces, which are modulated by the solution pH and the ionizable monomer position.

**Figure 7:**
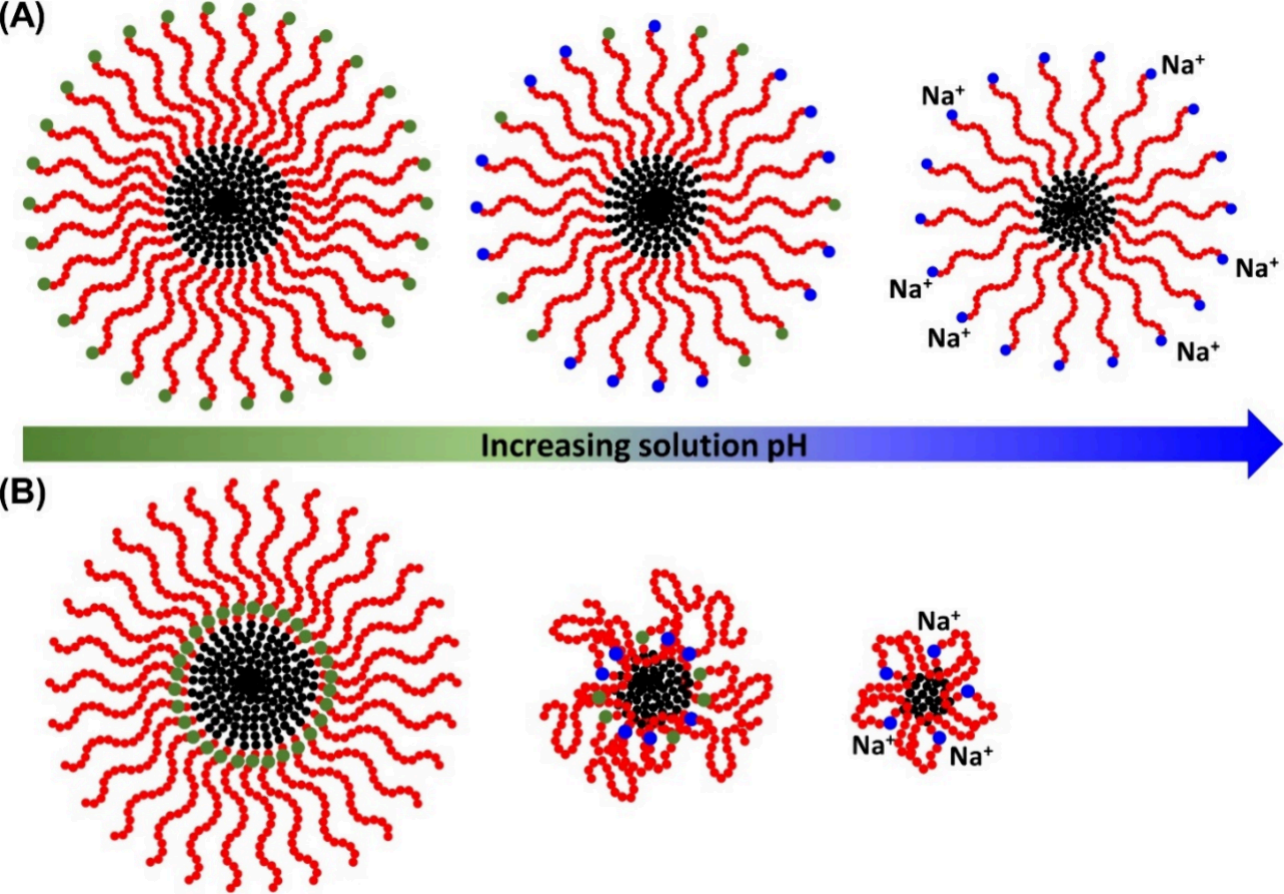
Schematic depiction of the structural changes undergone by micellar assemblies of sequence one micelles (A) and sequence three micelles (B) as a function of solution pH (Figure 1). The red and black circles represent the hydrophilic and hydrophobic monomers, respectively; the green and blue circles represent the neutral and ionized states of the ionized monomers (COOH vs COO^–^), respectively.

Figure 7 presents a depiction of our proposed micellar structure as a function of increasing solution pH. At the lowest solution pH value, where all ionizable CE monomers are expected to exist in the carboxylic acid form (COOH), all three sequences form similarly sized micelles. In this state, hydrophobic interactions and excluded volume interactions are expected to be the primary factors driving self-assembly as all ionic monomers remain charge-neutral, rendering contributions from electrostatic interactions negligible. As such, in this low pH regime, the minimization of interfacial tension between water and the hydrophobic segment is balanced by the excluded volume interactions of the hydrophilic segments to dictate the micellar structure. It is worth noting that hydrogen-bonding interactions involving COOH may also contribute to the low pH regime, potentially leading to intermicellar aggregation. This is supported by the occasional observation of elongated micelles in cryoTEM images (Figure 6A,D,G) and a broader micellar size distribution at low pH levels (Figures S8–S11). As the solution pH increases and the ionizable monomer begins to ionize, electrostatic interactions become a significant factor influencing the self-assembled structures of the peptoid BCP. At the low extent of ionization (i.e., ΔpH < 0), the micelles minimize the increasing electrostatic repulsion among polymer-bound ions by reducing the aggregation number and interfacial curvature (i.e., changing from sphere to ellipsoid). The electrostatic repulsion and excluded volume interaction are countered by the rising interfacial tension, which collectively dictates the micellar structure. By comparison, at the high extent of ionization (i.e., ΔpH ≥ 0), increasing electrostatic repulsion is mitigated by increasing counterion association with the micelles, preventing further decreases in aggregation number and micellar size.

The manner in which these micelles accommodate the increasing electrostatic repulsion with increasing solution pH is clearly influenced by the position of the ionic monomer along the polymer chain. If each sequence self-assembles into spherical core–shell-like aggregates of identical aggregation number, the proximity of neighboring ionized monomers increases as they are positioned deeper within the coronal interior (i.e., ionizable monomers in sequence three are closer to each other than those in sequence two, and the sequence two monomers are closer than those in sequence one). Therefore, as ionization increases, electrostatic repulsion within the aggregate also intensifies if no structural reorganization takes place. Sequence one accommodates increasing electrostatic repulsion by decreasing micellar aggregation number, resulting in the emergence of three distinct regimes across the studied pH range (Figure S6). In the first regime (RI, Figure S6A), when ΔpH < −1.25, there is no apparent change of micellar aggregation number and size with increasing pH; the sparse number of COO^–^ groups on the CE monomers remains sufficiently spaced to accommodate initial electrostatic repulsion. As ionization increases further with increasing pH, neighboring COO^–^ groups become too close to one another, prompting a decrease in the micellar aggregation number to alleviate the elevated electrostatic repulsion. This marks the beginning of regime two (RII, Figure S6A), where the micelles undergo notable and steady structural reorganization with increasing pH. For sequences where the ionizable monomer is repositioned into the coronal interior (sequences two and three), the first, pH-insensitive structural regime is absent. Instead, any increase in pH immediately initiates regime two (RII, Figure S6B). This is due to the reduced distance between neighboring ionizable monomers, where any increase in the COO^–^ content leads to significant electrostatic repulsion, causing reorganization at a lower pH.

In sequences two and three, there is a more pronounced decrease in micellar size throughout regime two compared to sequence one, which we attribute to more extensive chain reorganization within these micelles. Our previous work with this system revealed that at a solution pH of approximately 9, when CE monomers are fully ionized, the hydrophilic segment between the ionic monomer and the terminus of the hydrophilic block folds inward toward the micellar interior. This inward folding not only enhances favorable interactions between ionic monomers and the solvent but also minimizes the interfacial tension between water and the hydrophobic core by repositioning the hydrophilic segment to shield the core from unfavorable solvent interactions.

Once ΔpH ≥ 0, all three sequences reach a plateau in micellar sizes and aggregation numbers, which we define as regime three (RIII, Figure S6). In this regime, the micellization of the ionic peptoid BCPs and the resulting micellar structure are governed by the interplay of various interactions, including the hydrophobic effect, excluded volume interaction, electrostatic repulsion, and counterion association. At ΔpH ∼ 0, the ionic monomers are approximately 50% ionized. As the solution pH continues to increase, the extent of ionization is expected to reach near ∼100% by ΔpH ∼ 3. One might expect that the aggregation number and micellar size would continue to decrease with a further increase in pH if electrostatic repulsion is the dominant factor. However, despite the growing ionization level and the associated rise in charge density within the micellar aggregate, these micelles accommodate increasing electrostatic effects without any notable change in the aggregation number or micellar size. We attribute this stabilization in aggregation number and micellar size to the onset of counterion association: sodium cations (added during solution pH adjustments) become concentrated within the micellar boundary, effectively screening the electrostatic repulsion among the carboxylate groups (COO^–^) of the ionized CE monomers. This interpretation is supported by the lack of a continuous decline of zeta potential values of all micelle types when the solution pH exceeds p*K*_a_ (i.e., ΔpH ≥ 0). Increasing counterion association compensates for the otherwise intensifying electrostatic repulsion due to the greater extent of ionization.

## Conclusions

Our study has revealed that the micellar aggregation of these sequence-defined ionic peptoid BCPs can be controlled by a combination of the ionizable monomer position and solution pH. The way these micelles accommodate changes in the charge state of the ionizable monomer is strongly correlated to its position along the chain. Positioning the ionizable monomer at the terminus of the hydrophilic block produces micelles that are the least sensitive to changes in solution pH; these micelles exhibit a minimal decrease in micellar size and aggregation number and show an initial pH insensitivity at low pH values. In contrast, repositioning the ionizable monomer into the interior of the hydrophilic block results in micelles that respond more readily to pH changes and exhibit a greater overall reduction of micellar size and aggregation number as pH increases. We also observed that, at high pH, counterion associations between micelles and sodium cations become the primary mechanism to normalize increasing intramicellar charge density for all micellar types. These structural changes are governed by the interplay of hydrophobic effects, excluded volume interactions, and electrostatic interactions, all modulated by the position of the ionizable monomers along the chain. The findings of this study provide valuable insights into how molecular design, particularly the positioning of ionizable monomers, can be harnessed to create stimuli-responsive nanoassemblies capable of predictable and programmable structural reorganization.

## Supporting Information Available

Synthesis of peptoid block copolymers; MALDI-TOF MS and HPLC characterizations; and DLS, SANS, zeta potential, and cryoTEM sample analysis details (PDF)
