# Supplementary material for: Modulating the Aqueous Micellar Reorganization of Sequence-Defined Ionic Peptoid Block Copolymers by Ionizable Monomer Position and Solution pH
Source: Macromolecules. 2025 Feb 10;58(4):1851–8. doi: 10.1021/acs.macromol.4c02829 (PMC11866915; doi:10.1021/acs.macromol.4c02829)
Supplement: Supplementary file 1 — ma4c02829_si_001.pdf [file ma4c02829_si_001.pdf]

Supporting Information

Modulating the Aqueous Micellar Reorganization of Sequence-Defined Ionic Peptoid Block Copolymers by Ionizable Monomer Position and Solution pH

Bailee N. Barrett,[a,b] Pedram AziziHariri,[c] Vijay T. John [c] and Donghui Zhang [a,*]

[a,] *Department of Chemistry and Macromolecular Studies Group, Louisiana State University, Baton Rouge, LA 70803, United States*

[b,] *Current address: Department of Physics and Chemistry, Milwaukee School of Engineering, Milwaukee, WI 53202, United States.*

[c,] *Department of Chemical and Biomolecular Engineering, Tulane University, New Orleans, LA 70118, United States*

Corresponds to: dhzhang@lsu.edu

Table of Contents

1. Experimental Procedures S2
1.1. Materials S2
1.2. Synthesis of peptoid block copolymers S2
1.3. Matrix assisted laser desorption ionization-time-of-flight-mass spectrometry S4
1.4. High performance liquid chromatography S4
1.5. Dynamic light scattering S4
1.6. Small-angle neutron scattering S4
1.7. Zeta potential S4
1.8. Cryogenic transmission electron microscopy S5
2. Data Analysis Methods S5
2.1. Guinier analysis of small-angle neutron scattering data S5
3. References S15

1. Experimental Procedures

1.1. Materials. All solvents used were HPLC grade or comparable and were used as received. *N,N'*-dimethyldormamide, *N*-methylpyrrolidone, methylene chloride, triethylamine, trifluoroacetic acid, and acetic anhydride were purchased from Fisher Scientific. Rink amide resin, bromoacetic acid and *N,N'*-diisopropylcarbodiimide were purchased from ChemImpex Intl. *N*-decyl amine (>98%), 2-methoxyethylamine (≥98%), and 4-methylpiperidine (≥98%) were purchased from TCI. β-alanine t-butyl ester hydrochloride (95%) was obtained from Alfa Aesar. Hydrochloric acid (36.5-38%) and sodium hydroxide pellets (ACS) were obtained from BDH and AMRESCO, respectively. Deuterium oxide (99.9%) was purchased from Cambridge Isotope Laboratories.

1.2. Synthesis of peptoid block copolymers. A previously published solid-phase sub-monomer method procedure was used to synthesize the peptoid block copolymers on a Gyros Protein Technologies Prelude X peptide synthesizer.[S1] Briefly, 495 µmol Rink amide resin (0.51 meq./g) was swelled in DMF for 60 minutes (min) before deprotecting the Fmoc group with a 4-methylpiperidine solution (20% in *N*,*N*-dimethylformamide (DMF)) for 15 min. Acylation was achieved by reacting the amine functionalized resin with a mixture of bromoacetic acid (3 mL, 1.33 M in DMF, 8 eq.) and *N*,*N*'-diisopropylcarbodiimide (3 mL, 1.33 M in DMF, 8 eq.) at a 0.3 M total solution concentration for 5 min. The resin was then washed with DMF (3 × 10 mL). Subsequent nucleophilic displacement by the amine was done by mixing the bromoacylated resin with the appropriate amine [*i.e.*, 2-methoxyethylamine (3 mL, 2.67 M in DMF, 16 eq.), β-alanine OtBu ester HCl (6 mL, 1.33 M in DMF, 16 eq.), or *n*-decyl amine (3 mL, 2.67 M in *N*-methylpyrrolidone, 16 eq.)] as a 0.6 M solution in DMF for 10 min (for 2-methoxyethylamine and *n*-decyl amine residues) or 45 min (for β-alanine OtBu ester residues). Additionally, for the β-

alanine OtBu ester residues, triethylamine was added (6 mL, 3.6 M in DMF); upon completion of the reaction, the resin was mixed with chloroform (5 mL), followed by subsequent resin washes with DMF (10 mL), chloroform (3 × 10 mL), and DMF (3 × 20 mL). For all other residue additions, the resin was simply washed with DMF (3 × 10 mL) after displacement. Iterative acylation and displacement steps were performed until the desired peptoid chain length was achieved. At the conclusion of the synthesis, the resin was washed with methylene chloride (5 × 15 mL) and dried with nitrogen for 60 min. The crude peptoid block copolymers were cleaved from the resin in a 25% trifluoroacetic acid (TFA)/methylene chloride solution for 30 min. The resulting solution was evaporated under nitrogen to afford a viscous clear, yellow liquid. The *N*-terminus of peptoid block copolymers was end-capped by acylation at room temperature for 2 hours using acetic anhydride (~6 eq.) and triethylamine (added until solution was alkaline) in tetrahydrofuran at 0.07 M peptoid concentration. The solution was again evaporated under nitrogen to afford a viscous, clear yellow liquid. Purification of the crude product was achieved through dialysis against water followed by lyophilization to yield a white, fluffy powder.

Scheme S1.

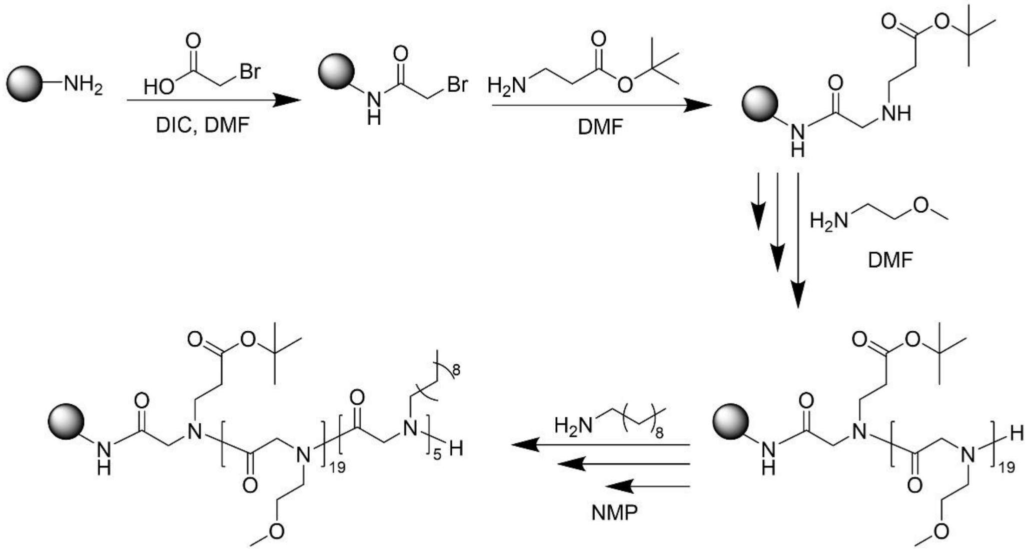

Scheme S2

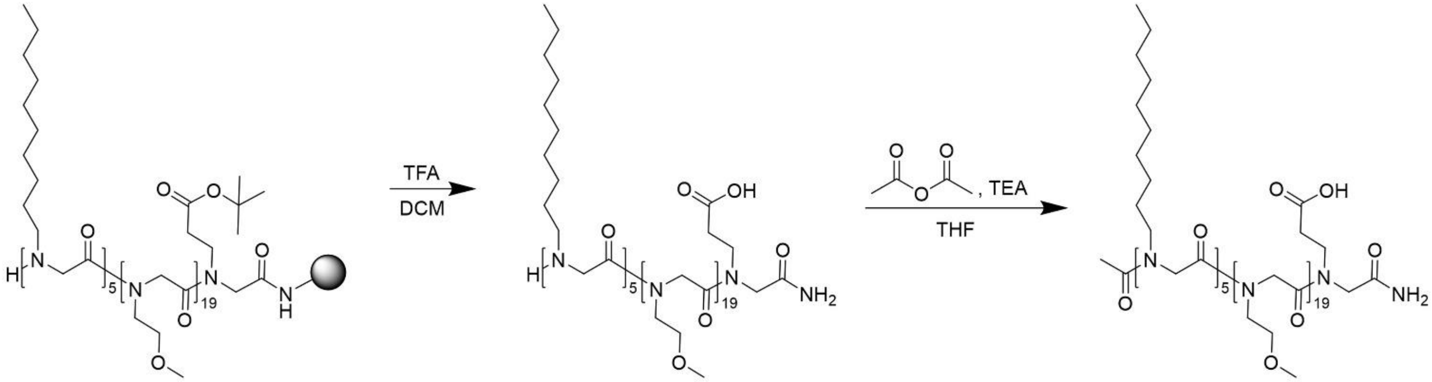

1.3. Matrix assisted laser desorption ionization-time-of-flight mass spectrometry (MALDI-TOF MS). MALDI-TOF MS measurements were conducted on a Bruker ultrafleXtreme tandem time-of-flight mass spectrometer equipped with a smartbeam-IITM 1000 Hz laser (Bruker Daltonics, Billerica, MA). Instrument calibration was achieved using Peptide Calibration Standard II (Bruker Daltonics, Billerica, MA). A saturated solution of α-cyano-4-hydroxycinnamic acid (CHCA) in methanol (MeOH) was used as the matrix. Spots for sample analysis were prepared by the successive deposition of sample solution (1 mg/mL in MeOH) and matrix (1 μL each) onto a 384-well ground steel sample plate. Each deposit was dried in air before subsequent depositions were made. A positive reflector mode was used for data acquisition and data analysis was completed using FlexAnalysis software. Representative MALDI-TOF MS spectra are shown in Figure S1.

1.4. High performance liquid chromatography (HPLC). HPLC analysis was performed with a Waters 616 pump, Waters 2707 Autosampler, and a 996 Photodiode Assay Detector which are controlled by Waters Empower 2 Software. Separation was performed on a Waters XSelect HSS Cyano column (3.5 µm, 75 x 3 mm) by a gradient resulting from mixing eluents A (0.1% TFA in water) and B (0.1% TFA in acetonitrile). The gradient ran from 40% B to 70% B in 30 min. The flow rate was 0.4 mL/min, and the detected wavelength was 215 nm. Samples were prepared for analysis by dissolving the peptoid block copolymers (0.5 mg/mL) in a 60% water/40% acetonitrile solvent mixture. Representative HPLC chromatograms are shown in Figure S2.

1.5. Dynamic light scattering (DLS). DLS measurements were conducted on a Wyatt Dawn Heleos-II instrument equipped with a 660 nm laser. Measurements were done at an angle of 99° and at a temperature of 25°C. Peptoid block copolymers were dissolved in pre-boiled ultrapure water (3 mg/mL) and the pH of the resulting solution was adjusted with either aqueous HCl solution (0.25 M) or aqueous NaOH solution (0.25 M), depending on the targeted pH value. Solutions were then heated in a water bath (70°C) for two hours and cooled to room temperature. Samples were filtered through PES syringe filters (0.45 µm) into scintillation vials for measurement. The hydrodynamic size was obtained from cumulants fitting of the decay curve.[S2]

1.6. Small-angle neutron scattering (SANS). SANS experiments were conducted at the Biological Small-Angle Neutron Scattering (Bio-SANS) instrument at the High Flux Isotope Reactor (HFIR) at Oak Ridge National Laboratory (ORNL) and NG7 30 m SANS instrument at the National Institute of Standards and Technology Center for Neutron Research (NCNR). Samples were prepared by dissolving the peptoid block copolymers in D_2O (3 mg/mL); the solution pH was subsequently adjusted using HCl or NaOH solutions (0.25 M in D_2O). The samples were then heated in a water bath (70°C) for two hours, cooled to room temperature, and then filtered through PES syringe filters (0.45 μm). Measurements were conducted in 2 mm Hellma banjo cells at 20°C for 75 min. Guinier analysis of the resulting absolute scattering intensity profiles was performed, yielding radius of gyration (R_g) and aggregation number (N_{agg}) for each micellar solution.

1.7. Zeta potential. Zeta potential measurements were conducted on a Malvern Zetasizer Nano ZS equipped with a 633 nm laser at 25°C. Peptoid block copolymers were dissolved in pre-boiled ultrapure water (3 mg/mL) and the pH of the resulting solution was adjusted with either aqueous

HCl solution (0.25 M) or aqueous NaOH solution (0.25 M), depending on the targeted pH value. Solutions were then heated in a water bath (70°C) for two hours and cooled to room temperature. Samples were filtered through PES syringe filters (0.45 µm) into clean folded capillary cells before measurement. Zeta potential values are reported as the average of three measurements for each sample.[S3, S4]

1.8. Cryogenic transmission electron microscopy (Cryo-TEM). Samples were prepared by dissolving peptoid block copolymers in pre-boiled ultrapure water (3 mg/mL for ΔpH ~ -3 samples and 10 mg/mL for ΔpH ~ 0 and 3 samples) and the pH of the resulting solution was adjusted with either aqueous HCl solution (0.25 M) or aqueous NaOH solution (0.25 M), depending on the targeted pH value. Solutions were then heated in a water bath (70°C) for two hours and cooled to room temperature. Samples were filtered through PES syringe filters (0.45 µm) before imaging. For imaging, samples were prepared under a controlled environment, using an FEI Vitrobot. After mounting a 200-mesh lacey carbon-coated copper grid (TED PELLA, INC), 5 µL of the sample was dispersed on the grid using a pipette. The excess sample was then removed by blotting the grid with filter papers attached to the arms of the vitrobot for two seconds, leaving behind a thin sample film. The film was then vitrified quickly by plunging into liquid ethane, followed by liquid nitrogen. Finally, the samples were transferred onto a single-tilt cryo-specimen holder for imaging. All the images were taken below -170°C with an FEI G2 F30 Tecnai TEM operated at 100kV.

2. Data Analysis Method

2.1. Guinier analysis of small-angle neutron scattering data. Equations S1 and S2 were used to perform Guinier analysis of the measured absolute intensity profile for each of the sample solutions.[S5]

$$I(Q) = I_0 \exp\left(-\frac{1}{3}Q^2R_g^2\right), \qquad \text{(S1)}$$

Where $I(Q)$ is the measured scattering intensity, $I_0 = nV_{micelle}^2(\rho_{D_2O} - \rho_{chain})^2$ (n is the number density micelle, $V_{micelle}$ is the micellar volume, ρ_{D_2O} is the scattering length density of D_2O, and ρ_{chain} is the scattering length density of a peptoid chain), Q is the momentum transfer vector, and R_g is the radius of gyration.

$$N_{agg} = \frac{I_{0,measured}}{(\phi)(V)^2(\rho_{D_2O} - \rho_{chain})^2}, \qquad \text{(S2)}$$

Where N_{agg} is the aggregation number, $I_{0,measured}$ is the intensity of $I(Q)$ at $Q \to 0$, ϕ is the sample volume fraction, V is the volume occupied by a singular peptoid chain, and ρ_{D_2O} is the scattering length density of D_2O, and ρ_{chain} is the scattering length density of a peptoid chain. The Q-range selected for analysis was chosen such that $R_g \times Q < 1$, and the scattering length densities (SLDs) of the hydrophobic and hydrophilic blocks were calculated from previously reported mass densities.[S6]

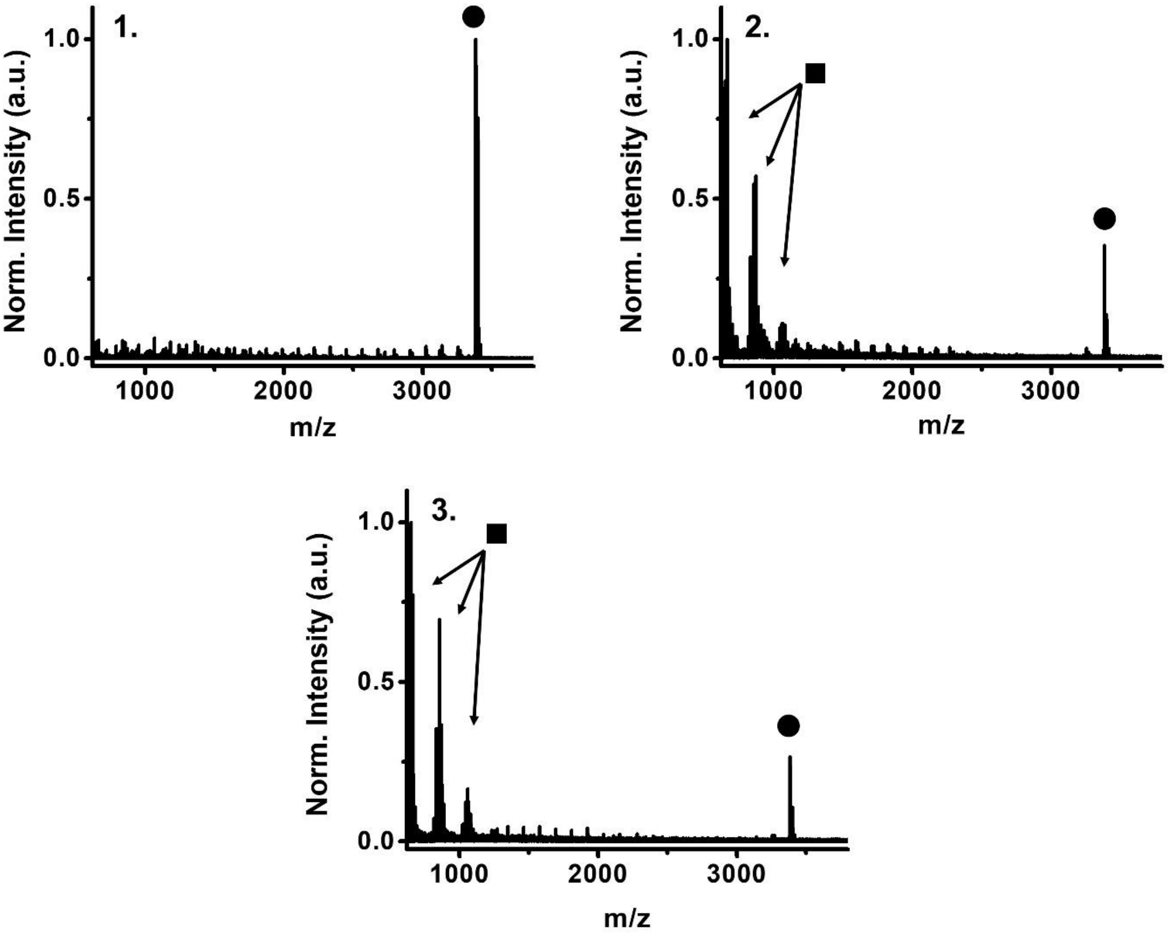

Figure S1. Representative MALDI-TOF MS spectra of sequence-defined peptoid block copolymers (Sequence one, two and three, Figure 1B). Peaks signified by the filled circles and squares correspond to targeted peptoid block copolymers and the matrix molecules, respectively.

Table S1. Experimentally determined exact molecular weights of various sequence-defined peptoid block copolymers (Sequence 1-3, Figure 1B) by the MALDI-TOF MS analysis, the calculated exact molecular weights based on molecular formula and the sample purity level based on HPLC analysis.

Sequence #	Molecular Formula		Calc. (m/z)	Found (m/z)	Purity Level (%)
One	$C_{162}H_{298}N_{26}O_{47}Na$	$[M+Na]^+$	3383.162	3383.25	>99
Two	$C_{162}H_{298}N_{26}O_{47}Na$	$[M+Na]^+$	3383.162	3383.31	>99
Three	$C_{162}H_{298}N_{26}O_{47}Na$	$[M+Na]^+$	3383.162	3383.31	>99

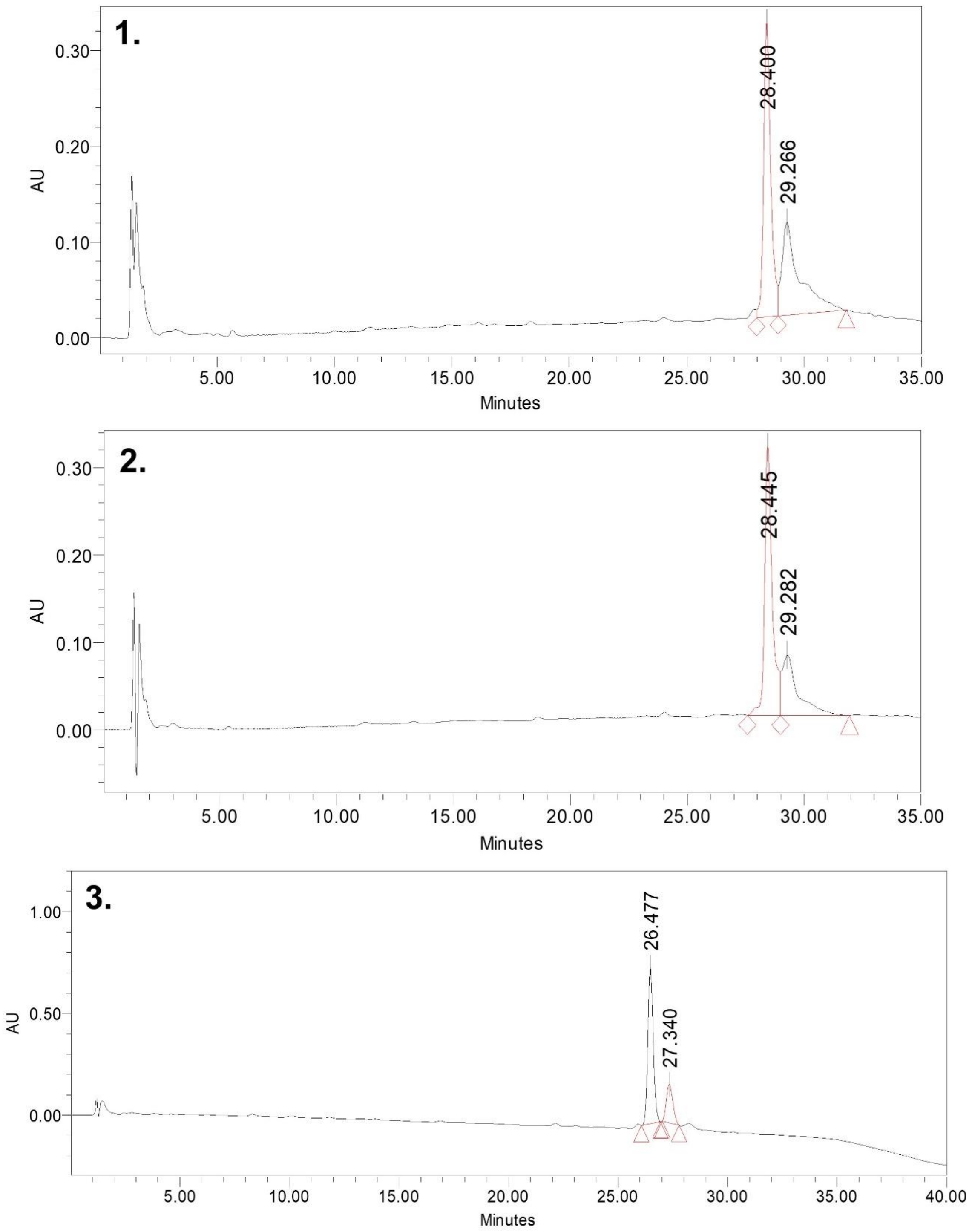

Figure S2. Representative HPLC chromatograms of sequence-defined peptoid block copolymers (Sequence one, two and three, Figure 1B). Note that the peaks eluted out in the 26-30 min range all correspond to the full length peptoid block copolymer as verified by MALDI-TOF MS analysis.

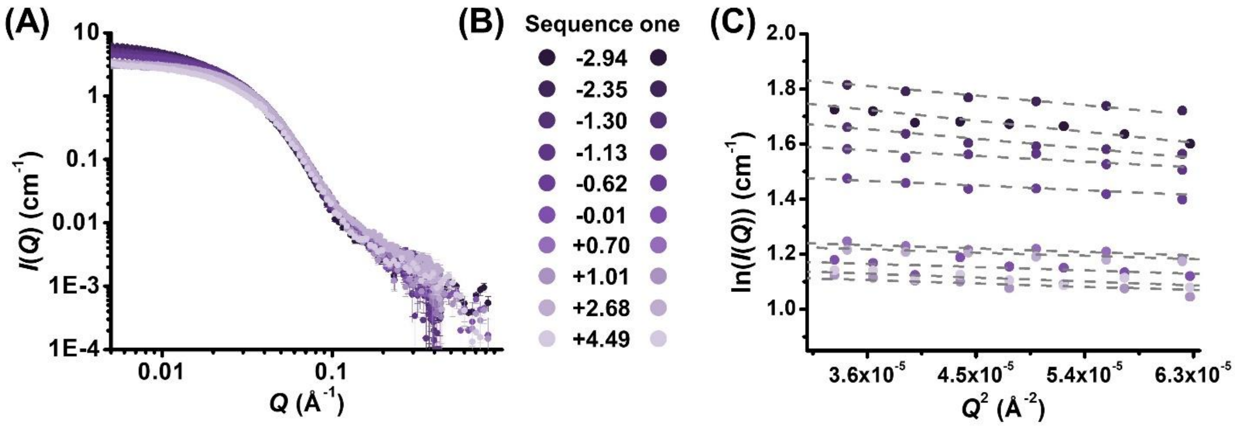

Figure S3. (A) Absolute scattering intensity profiles of sequence one ionic sequence-defined peptoid BCP micelles (Figure 1) at all ten solution ΔpH values; (B) Legend of ΔpH values and corresponding color shades for both the absolute scattering profiles and Guinier plot regions; decreasing color intensity represents higher solution pH (*i.e.*, higher values of ΔpH); (C) Representative region of the Guinier plot for sequence one micellar solutions (Figure 1) at all ten measured solution ΔpH values; the grey dashed line represents the linear fit of the data.

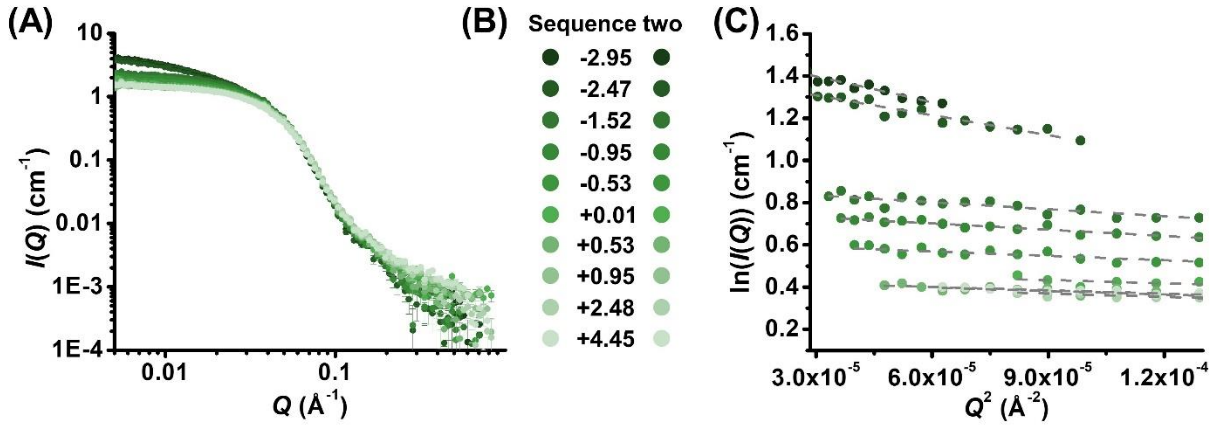

Figure S4. (A) Absolute scattering intensity profiles of sequence two ionic sequence-defined peptoid BCP micelles (Figure 1) at all ten solution ΔpH values; (B) Legend of ΔpH values and corresponding color shades for both the absolute scattering profiles and Guinier plot regions; decreasing color intensity represents higher solution pH (*i.e.*, higher values of ΔpH); (C) Representative region of the Guinier plot for sequence two micellar solutions (Figure 1) at all ten measured solution ΔpH values; the grey dashed line represents the linear fit of the data.

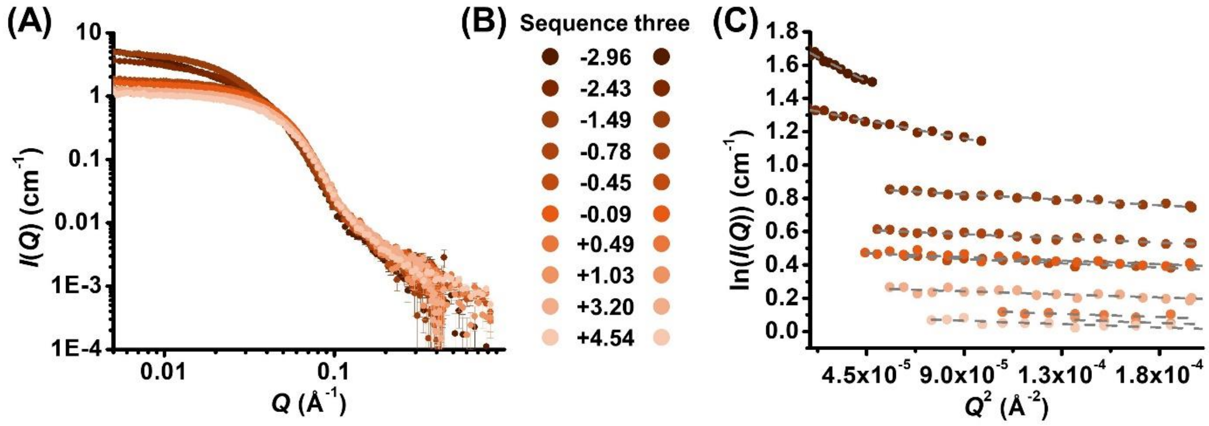

Figure S5. (A) Absolute scattering intensity profiles of sequence three ionic sequence-defined peptoid BCP micelles (Figure 1) at all ten solution ΔpH values; (B) Legend of ΔpH values and corresponding color shades for both the absolute scattering profiles and Guinier plot regions; decreasing color intensity represents higher solution pH (*i.e.*, higher values of ΔpH); (C) Representative region of the Guinier plot for sequence three micellar solutions (Figure 1) at all ten measured solution ΔpH values; the grey dashed line represents the linear fit of the data.

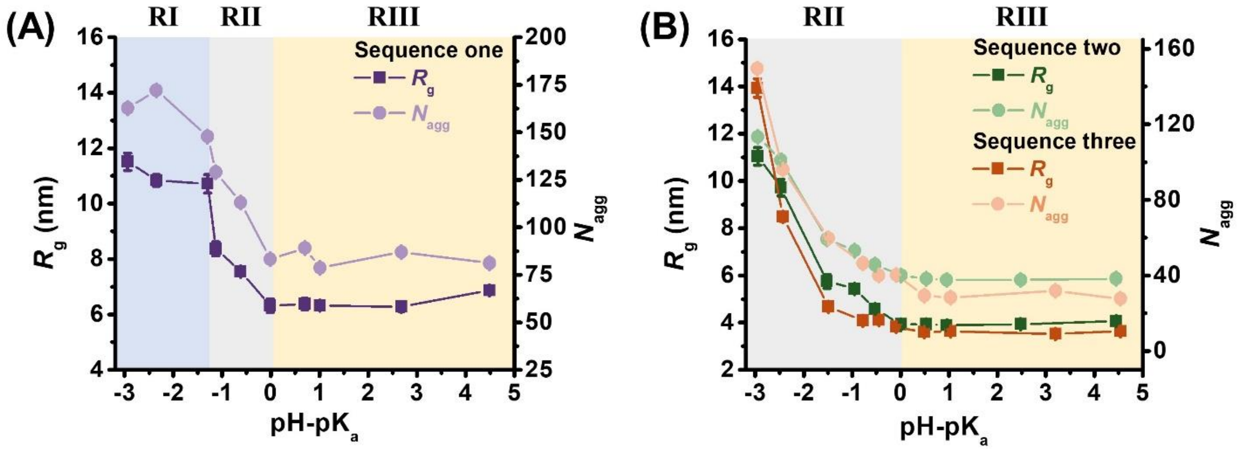

Figure S6. (A) Radius of gyration and aggregation number as a function of pH-pK_a for sequence one micelles with regime one, (RI, blue shading), regime two (RII, grey shading), and regime three (RIII, yellow shading) indicated; (B) Radius of gyration and aggregation number as a function of pH-pK_a for sequences two and three micelles with regime two (RII, grey shading) and regime three (RIII, yellow shading) indicated.

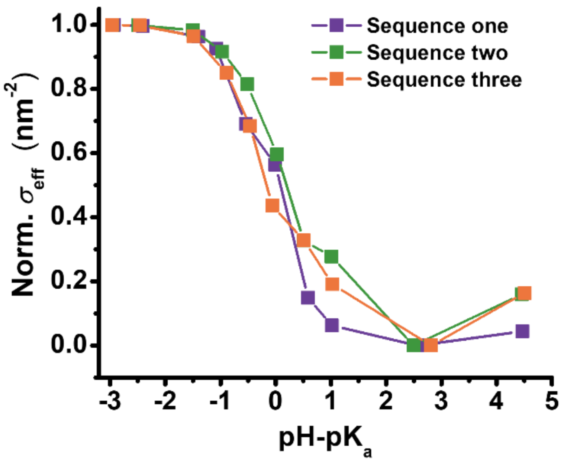

Figure S7. Plots of normalized effective surface charge density as a function of pH-pK$_a$ (ΔpH; *i.e.*, increasing ΔpH corresponds to increasing solution pH) for the peptoid BCP micelles.

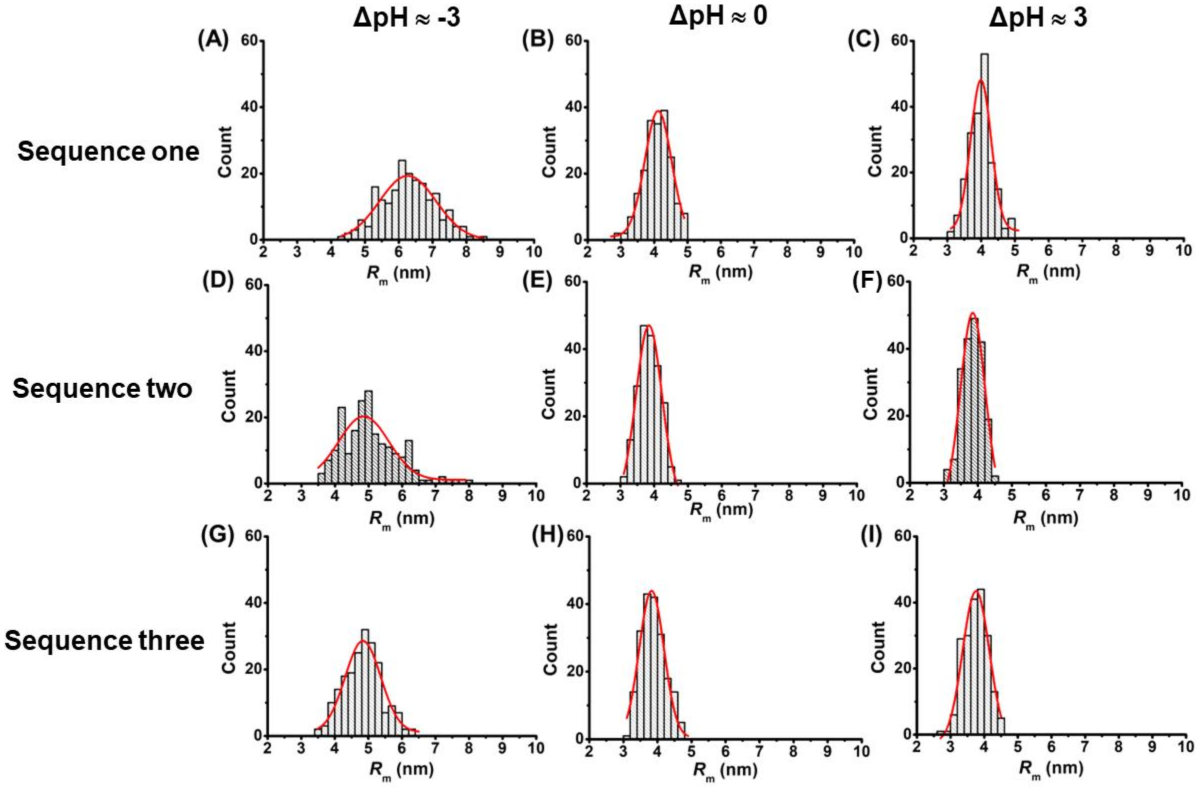

Figure S8. Histogram analysis showing the micellar radius (R_m) distribution of ionic sequence-defined peptoid BCP micelles (sequence one (A-C), sequence two (D-H), sequence 3 (G-I), Figure 1) at three different ΔpH value (*i.e.*, ΔpH ≈ -3, 0 and 3). The R_m of 200 micelles in the cryoTEM images (Figure 6) were measured using ImageJ software.

Table S2. Micellar radius (R_m), micellar aspect ratio, and radius of gyration (R_g) of sequence one, two and three peptoid micelles in aqueous solutions of various pH.

~ΔpH	Sequence one			Sequence two			Sequence three		
	R_g [a] (nm)	R_m [b] (nm)	Aspect Ratio [b]	R_g [a] (nm)	R_m [b] (nm)	Aspect Ratio [b]	R_g [a] (nm)	R_m [b] (nm)	Aspect Ratio [b]
-3	11.5 ± 0.3	6.3 ± 0.8	1.2 ± 0.2	11.0 ± 0.4	5.0 ± 0.8	1.3 ± 0.2	13.9 ± 0.4	4.8 ± 0.6	1.3 ± 0.2
0	6.3 ± 0.2	4.1 ± 0.4	--	3.9 ± 0.1	3.8 ± 0.3	--	3.82 ± 0.04	3.9 ± 0.4	--
3	6.3 ± 0.2	4.0 ± 0.4	--	3.93 ± 0.07	3.8 ± 0.3	--	3.52 ± 0.05	3.7 ± 0.3	--

[a] R_g was determined by Guinier analysis of the SANS profile; [b.] R_m and aspect ratio of non-spherical micelles were determined by cryoTEM analysis.

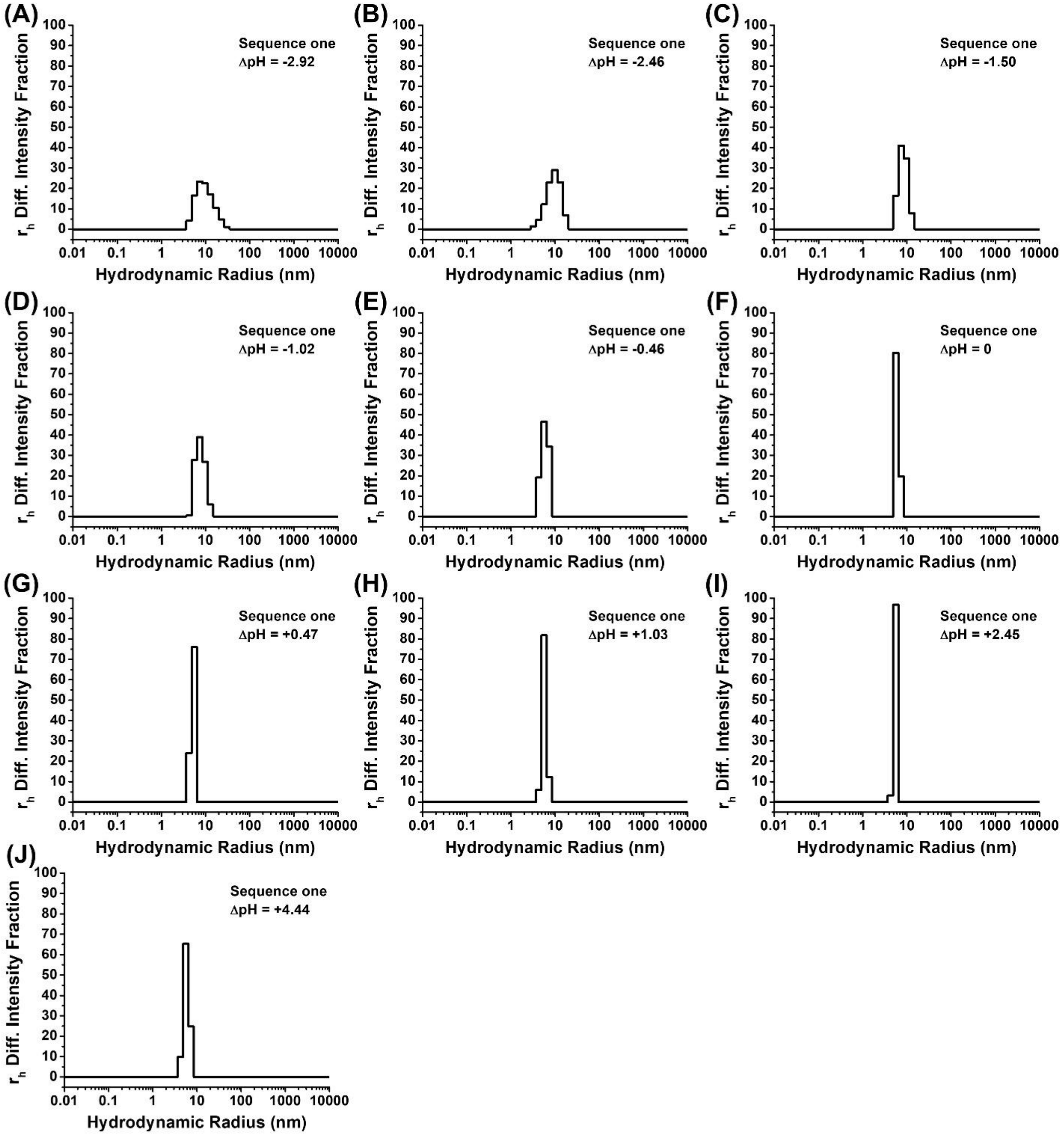

Figure S9. Intensity weighted size distribution of sequence one DLS samples. Regularization method was used to obtain the distribution.

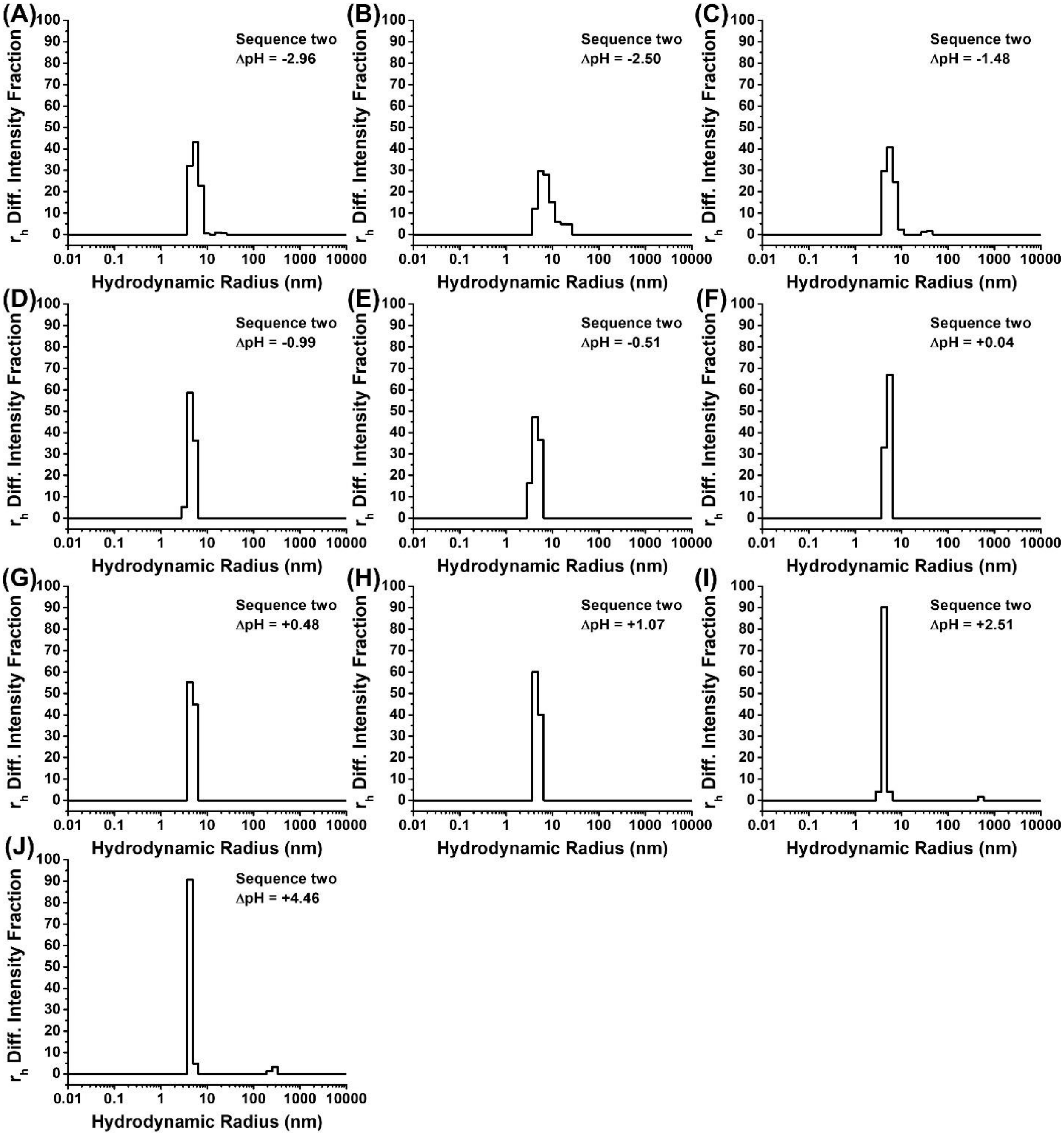

Figure S10. Intensity weighted size distribution of sequence two DLS samples. Regularization method was used to obtain the distribution.

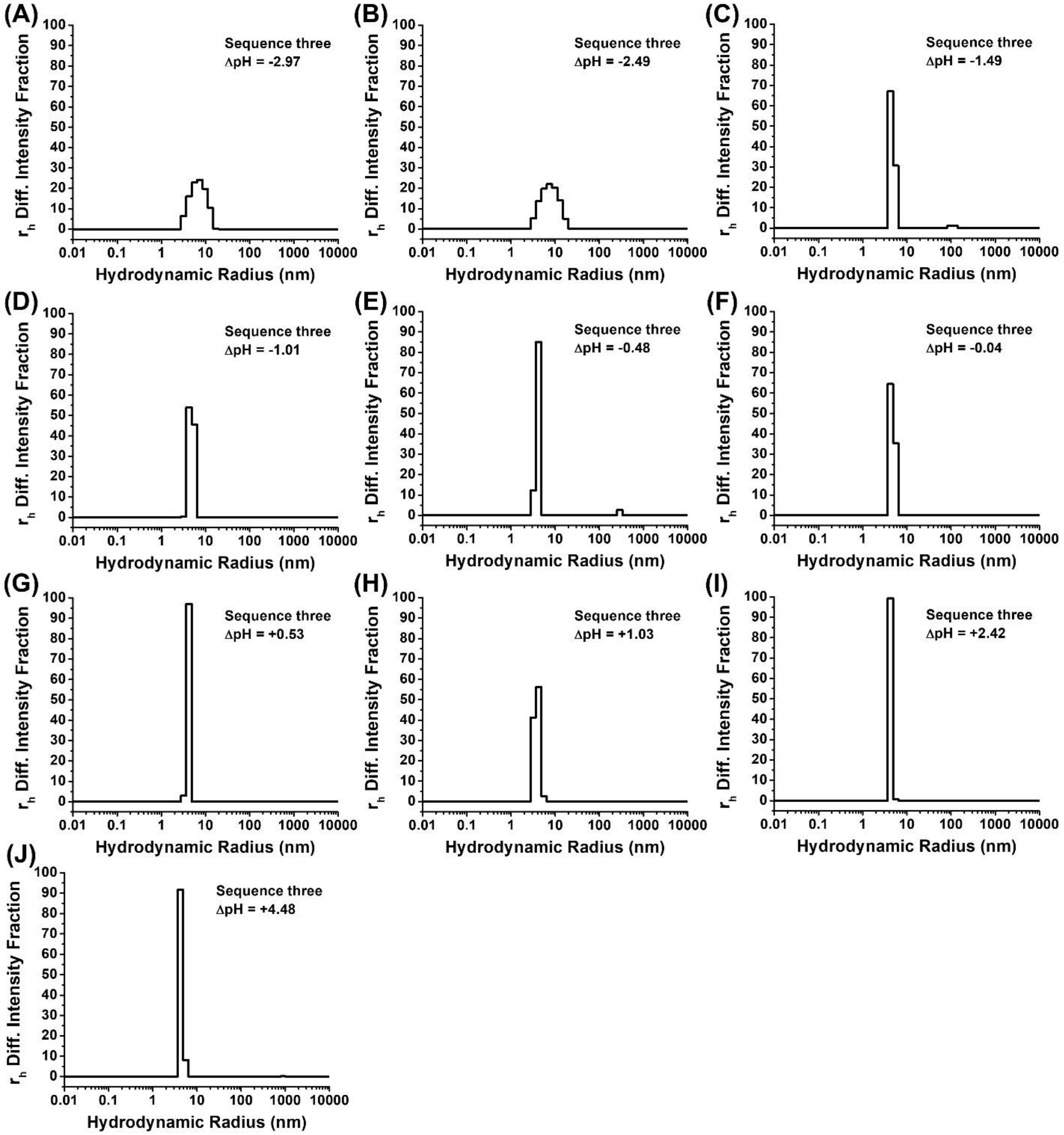

Figure S11. Intensity weighted size distribution of sequence three DLS samples. Regularization method was used to obtain the distribution.

3. References

(S1) Barrett, B. N.; Tung, C.-H.; Huang, G.-R.; Hossain, I.; Do, C.-W.; John, V. T.; Chen, W.-R.; Zhang, D. Modulating Water Distribution and the Intramicellar Assembly of Sequence-Defined Ionic Peptoid Block Copolymers by the Ionic Monomer Position. *Macromolecules* **2023**, *56* (14), 5306-5313.

(S2) Koppel, D. E. Analysis of Macromolecular Polydispersity in Intensity Correlation Spectroscopy: The Method of Cumulants. *J. Chem. Phys.* **2003**, *57* (11), 4814-4820.

(S3) O'Brien, R. W.; White, L. R. Electrophoretic mobility of a spherical colloidal particle. *J. Chem. Soc. Faraday Trans.2: Mol. Chem. Phys.* **1978**, *74* (0), 1607-1626.

(S4) Tscharnuter, W. W. Mobility measurements by phase analysis. *Appl. Opt.* **2001**, *40* (24), 3995-4003.

(S5) Nayem, J.; Zhang, Z.; Tomlinson, A.; Zarraga, I. E.; Wagner, N. J.; Liu, Y. Micellar Morphology of Polysorbate 20 and 80 and Their Ester Fractions in Solution via Small-Angle Neutron Scattering. *J. Pharm. Sci.* **2020**, *109* (4), 1498-1508.

(S6) Murnen, H. K.; Rosales, A. M.; Dobrynin, A. V.; Zuckermann, R. N.; Segalman, R. A. Persistence length of polyelectrolytes with precisely located charges. *Soft Matter* **2013**, *9* (1), 90-98.
